# HAL fluorescence cystoscopy and TURB.One year of Romanian experience

**Published:** 2009-04-25

**Authors:** B Geavlete, R Mulţescu, D Georgescu, M Jecu, P Geavlete

**Affiliations:** ‘Sf. Ioan’ Clinical Emergency Hospital, Department of Urology, BucharestRomania

**Keywords:** hexaminolevulinate, blue light cystoscopy, blue light transurethral bladder resection, non-invasive bladder tumors, recurrence rate, false-positive results

## Abstract

Hexaminolevulinate blue light cystoscopy (HAL–BLC) represents an increasingly acknowledged method in bladder cancer diagnostic. We aimed to evaluate the importance of this procedure in cases of non–invasive bladder tumors (NIBT), to compare it with standard white light cystoscopy (WLC), and to establish the efficiency of blue light transurethral bladder resection (BL–TURB).

Between December 2007 and December 2008, WLC and BLC were performed in 70 cases. WL–TURB was performed for all lesions visible in WL, and BL–TURB for those only detected in BL. Patients diagnosed with NIBT were followed–up after an average period of 5 months (between 18 and 22 weeks) by WLC and BLC. The control group included the same number of consecutive cases of NIBT, which underwent only WLC and WL–TURB, as well as the same follow–up protocol as the study group.

WLC correctly identified 115 tumors, and BLC, 157 tumors. The detection rate was 68.8% for WLC, with a 9.4% rate of false–positive results, and 94% for BLC, with a 14.6% rate of false–positive results. The diagnostic accuracy in CIS lesions was 57.3% for WLC and 95% for BLC, while in pTa tumors; it was 68.8% for WLC and 94% for BLC. 62 cases of the study group diagnosed with NIBT emphasized a recurrence rate of 6.4% after 5 months. The control group described a recurrence rate of 24.2%.

HAL fluorescence cystoscopy is a valuable diagnostic method for patients with NIBT, with considerably improved accuracy by comparison to WLC, and a significant impact upon the short–term recurrence rate.

## Introduction

Bladder cancer represents a common malignancy, with a severely high recurrence rate. Despite the fact that WLC is still regarded as thegold–standard diagnostic method for NIBT [1], small papillary (Ta, T1) and especially flat (carcinoma in situ–CIS) urothelial lesions can be easily overlooked, thus leading to a significant increase of the recurrence rate [2].

Therefore, while searching for a more sensitive diagnostic tool, the photodynamic diagnosis (PDD) emerged as a promising solution.

The aminolevulinic acid (ALA) was among the first products used for PDD. After introducing its improved ester, the hexyl aminolevulinate (HAL–Hexvix), PDD acquired a substantial acknowledgement in practical use.

The EAU Guidelines state that fluorescence cystoscopy, performed in blue light (BL) and using a porphyrin–based photosensitizer, may help discovering suspicious areas, not visible in white light (WL) [1].

Moreover, BL–TURB can be expected to improve the quality of the endoscopic treatment, allowing a more complete resection of the existing tumors.

In this study, we aimed to establish the value of HAL–BLC as complementary diagnostic tool in patients suspected of bladder cancer, to compare it with standard cystoscopy, and to clarify the advantages of BL–TURB over the standard resection. We also attempted to determine the influence of this diagnostic and treatment method over the short–term recurrence rate in such cases.

## Materials and Methods

Between December 2007 and December 2008, 70 consecutive cases (49 men and 21 women), with a mean age of 66 years (range 34 to 83), have been admitted and investigated for hematuria and/or positive urinary cytology.

A standard investigative protocol which included general clinical examination, blood tests, urine culture, abdominal ultrasonography, IVP and eventually a CT scan was applied in all cases.

The exclusion criteria included massive hematuria (BL being highly absorbed by blood and clots), moderate to severe leucocyturia, and prior history of bladder cancer or imagistic evidence of upper urinary tract disease.

HAL bladder instillation (100mg dissolved in 50mL phosphate buffer solution, 8mmol) was performed using a 14Ch bladder catheter, after complete voiding, 1 to 2 hours prior to cystoscopy (an average instillation time of 90 minutes).

The Storz D–light–C system with a xenon arc lamp as source was used in all cases.

The first stage consisted of careful WLC, resulting in a bladder map of the suspicious lesions, followed by BLC, which described a specific map of all distinctive fluorescent areas.

WL–TURB was performed for all the suspected lesions identified by WLC, while BL–TURB was applied for tumors discovered only by BLC. All suspicious areas were entirely resected.

The histological analysis emphasized a ‘pathological’ bladder chart for each patient. We compared the 3 bladder maps in order to determine the accuracy of these diagnostic methods.

Patients diagnosed with NIBT were followed after an average period of 5 months (between 18 and 22 weeks) by both WLC and BLC, and underwent WL and eventually BL–TURB for all suspicious areas.

The control group included the same number of consecutive patients with NIBT, initially diagnosed only by WLC, which underwent WL–TURB, as well as the same intravesical therapy and follow-up protocol as the study group. The recurrence rates were compared between the 2 groups.

## Results

The cystoscopic and pathological results described 4 different groups of patients.

**Fig 1:**
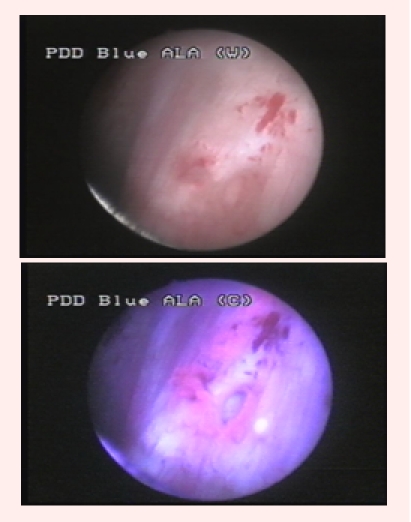
pT1G1 urothelial bladder tumor visible both in WLC (left) and BLC (right)

Group I consisted of patients with identical bladder maps according to WLC and BLC, and entirely confirmed by the pathological examination. This group included 31 cases (44.2%) with 73 tumors identified by both diagnostic tools (23 CIS, 32 pTa, 15 pT1 and 3 pT2) (Fig 1).

**Fig 2:**
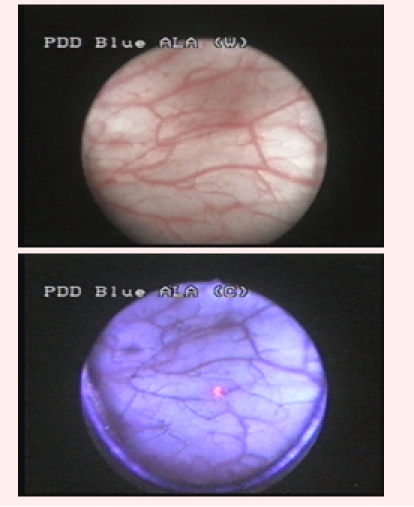
Distinctive fluorescent aspect of a pTaG1 tumor, not visible in white light

Group 2 was represented by 26 patients (37.1%) diagnosed with bladder cancer by WLC, and in which BLC showed at least one supplementary bladder tumor. Overall, WLC described 48 suspicious lesions, 42 being pathologically confirmed (12 CIS, 16 pTa, 12 pT1 and 2 pT2). BLC identified 43 additional lesions, 33 of which being confirmed by pathology (16 CIS and 17 pTa) (Fig 2).

**Fig 3:**
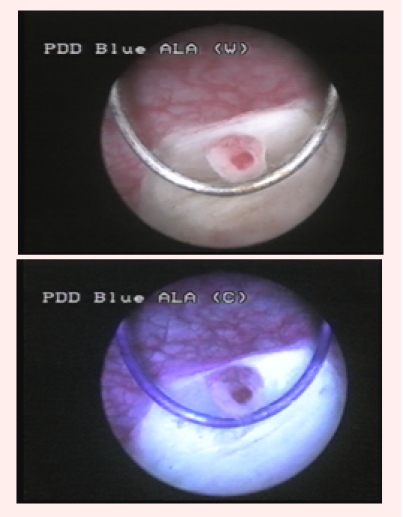
pTaG1 tumor, visible in white light, with no fluorescence in blue light

In total, BLC described 65 tumors (25 CIS, 26 pTa, 12 pT1 and 2 pT2), while 17 fluorescent lesions had no pathological confirmation. In 5 cases, 10 malignancies (3 CIS and 7 pTa) described by WLC presented no fluorescence in BLC (Fig 3).

**Fig 4:**
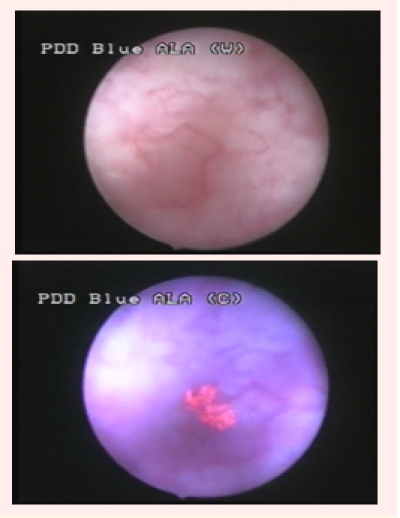
CIS lesion, visible only in blue light

Group 3 consisted of 10 patients (14.2%) tumor–free in WL, but in which BLC described 29 fluorescent areas, 19 being confirmed by pathology as malignant (10 CIS and 9 pTa) (Fig 4).

**Fig 5:**
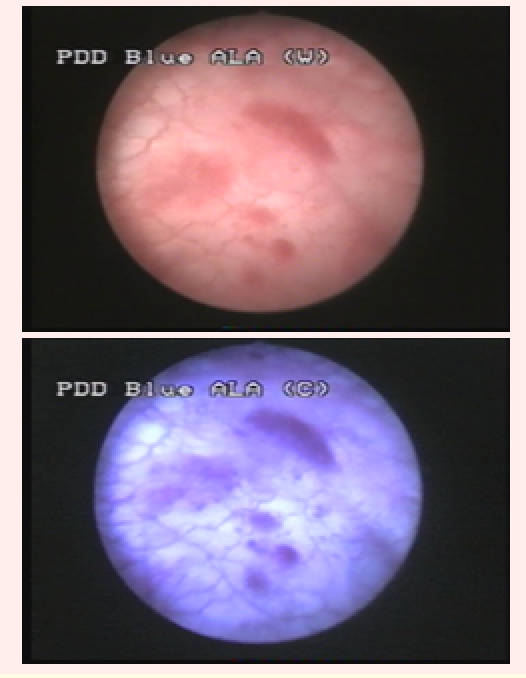
Suspicious flat lesion in white light, with no fluorescence in blue light and no pathological confirmation

Group 4 included 3 cases (4.2%) with 6 apparently malignant lesions according to WLC, no fluorescence in BLC and no pathological confirmation (Fig 5).

In total, a number of 167 tumors (61 CIS, 74 pTa, 27 pT1 and 5 pT2) were diagnosed in 67 cases (95.7%). BLC provided additional diagnostic information in 36 patients (51.4%), describing 52 supplementary tumors (26 CIS and 26 pTa–31.1%) by comparison to WLC, which only found 10 additional lesions (3 CIS and 7 pTa–5.9%).

The sensitivity of WLC was 68.8% (115 correctly diagnosed tumors – 35 CIS, 48 pTa, 27 pT1, 5 pT2), and the rate of false-positive results was 9.4% (12 negative biopsies).

The diagnostic accuracy of BLC was 94% (157 confirmed tumors – 58 CIS, 67 pTa, 27 pT1, 5 pT2), while the rate of false–positive results was 14.6% (27 negative biopsies).

**Fig 6:**
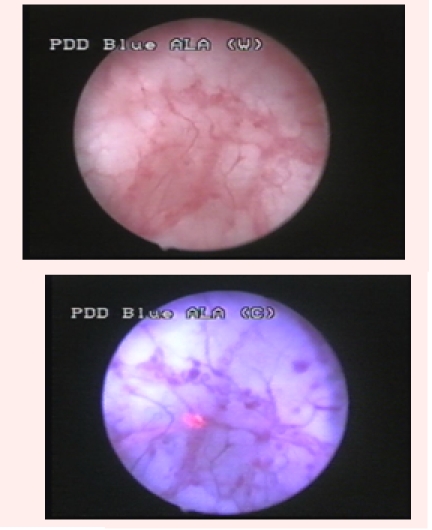
CIS lesion not visible in white light but fluorescent in blue light

The most relevant differences in detection rates occurred for CIS lesions, 95% of them being correctly diagnosed by BLC, and only 57.3% by WLC (Fig 6).

Differences remained significant concerning the pTa tumors, for which WLC emphasized a diagnostic accuracy of 64.8%, and BLC, of 90.5%.

**Fig 7:**
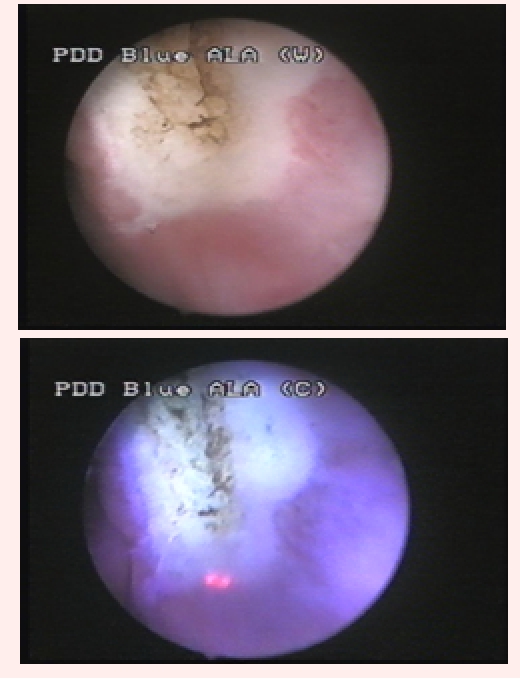
Fluorescent margin of a pT1G2 tumor after WL–TURB, confirmed by pathology

In 7 of the 67 cases of bladder cancer (10.4%), malignant lesions diagnosed by WLC (1 pTa, 5 pT1 and 1 pT2 tumor) emphasized after WL–TURB fluorescent positive margins in blue light, confirmed by pathology (Fig 7).

There were no specific complications related to HAL–BLC.

After an average period of 5 months, WLC, BLC and eventually re–TURB were performed for the 62 patients of the study group initially diagnosed with NIBT, as well as for the similar number of patients included in the control group.

The tumor recurrence rate was 6.4% (4 cases with 3 CIS and 3 pTa tumors) for the study group and significantly higher, 24.2% (15 cases with 12 CIS and 10 pTa), for the control group.

## Commentaries

The basis of HAL–BLC is represented by the preferential accumulation of the photoactive porphyrin in the neoplastic tissue, resulting in red fluorescence emitting tumors [3]. By comparison to ALA, HAL provides the benefits of increased selectivity, brighter fluorescence and shorter instillation time [4,5,6].

The prostate urethra, bladder neck, ureteral orifices, as well as the tangential view of the bladder mucosa may create aspects of false fluorescence, and therefore the bladder must be fully distended and lesions should be directly illuminated. Lesions in which fluorescence fades by pressing the concerned area with the resection loop are usually benign and resection is unnecessary.

Bladder inflammation may determine increased fluorescence of the mucosa and consequently, false–positive results. BLC should not be performed any sooner than 6 weeks after BCG (Bacillus Calmette–Guerin) intravesical instillations. Our protocol included a minimum period of 2 months between the last instillation and the follow–up cystoscopic control.

On the other hand, BL is highly absorbed by blood and clots. Therefore, resection related bleeding may result in poor visualization and reduced diagnostic accuracy.

According to a study performed by Jichlinski et al., BLC is well tolerated, with no side effects related to HAL bladder instillation [7].

The diagnostic accuracy in our series was significantly improved for BLC (94%) by comparison to WLC (68.8%). The literature data mention quite resembling results, specifically 97% versus 78% [4], and 96% versus 77% [8].

Groups II and III clearly emphasized the advantages provided by this type of cystoscopy. It is highly significant that in 51.4% of the cases, HAL–BLC diagnosed at least one additional bladder tumor. A European phase 3 multicenter study on 211 patients obtained a similar rate, describing supplementary tumors in 55% of cases [4].

**Fig 8:**
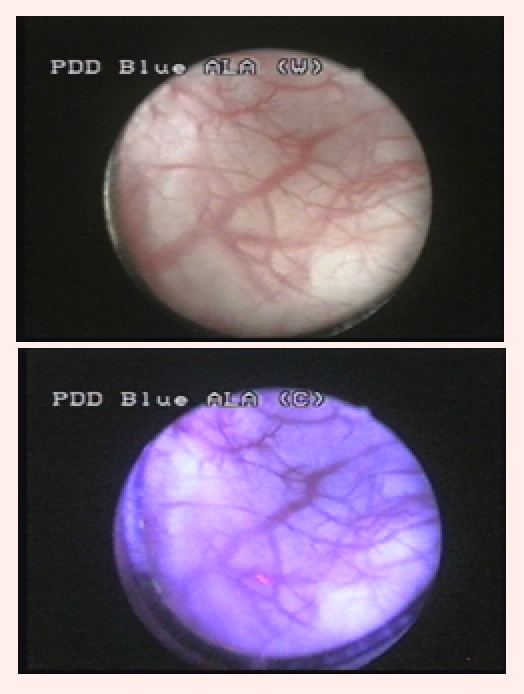
CIS lesion diagnosed only by blue light cystoscopy

More so, it is remarkable that 14.2% of our patients were diagnosed with bladder cancer only by BLC and declared cancer–free by WLC ( Fig 8).

**Fig 9:**
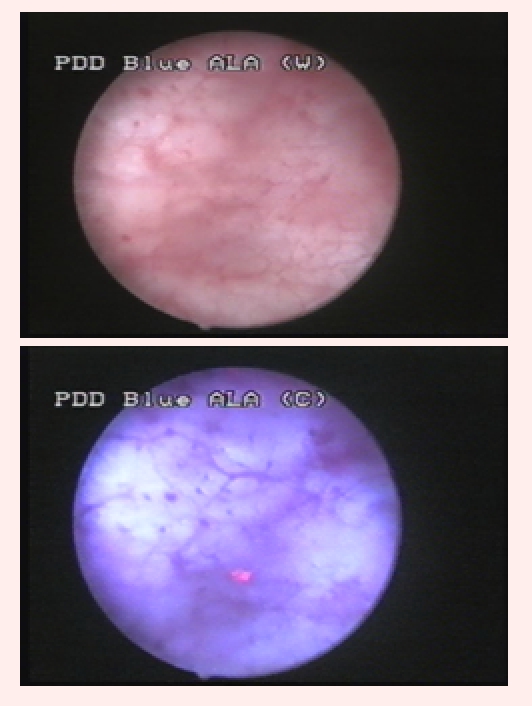
CIS lesion omitted by white light

Our study emphasized a CIS detection rate of 95% for BLC, significantly superior to WLC, which only diagnosed 57.3% of the CIS lesions (Fig 9).

Fradet et al. obtained comparable results in an American phase 3 multicenter study on 298 patients, which described a CIS detection rate of 92% for BLC and 68% for WLC, respectively [3]. Schmidbauer et al. [4] determined results closer to ours, showing a CIS detection rate of 97% for BLC, and of 58% for WLC.

**Fig 10:**
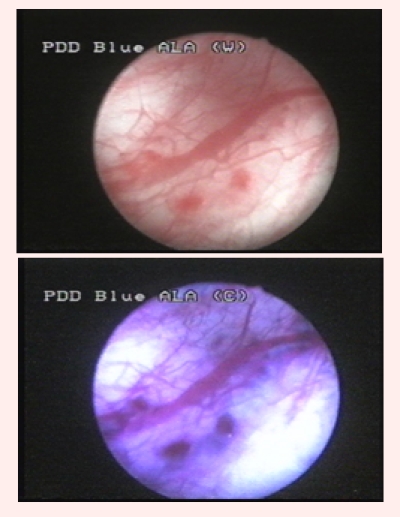
Suspicious lesion in white light, with no fluorescence and no pathological confirmation

We emphasized a 9.4% rate of false–positive results for WLC, and of 14.6% for BLC (Fig 10).

The study by Schmidbauer et al. showed similar data (10% for WLC versus 13% by BLC) [4]. However, Grossman et al. [9] and Jocham et al. [8] presented higher false–positive rates (39% versus 31% and 37% versus 26%, respectively).

It was proved that recurrence at any site of the bladder during the first follow–up cystoscopy after TUR constitutes one of the most important prognostic factors for time to progression. Consequently, PDD might be most useful in patients with primary tumors [10].

It is quite obvious that tumors shortly diagnosed after TURB are mostly lesions overlooked during the primary procedure rather than newly developed malignancies. This is the rationale for trying to improve the recurrence rate and prognostic of the patients by superior diagnostic accuracy.

Among our cases, the recurrence rate at 5 months was 6.4% for the BLC group and 24.2% for the WLC group.

Similarly, the comparative analysis performed by Denziger et al. on 191 cases with NIBT, diagnosed either by WLC or BLC, described a residual tumors' rate at 6 weeks after primary TURB of 25.2% and 4.5%, respectively.The recurrence rates among these patients after an average follow–up period of 86 months were 44% in the WLC arm versus 16% in the BLC arm [11].

Daniltcenko et al. emphasized the fact that differences between the recurrence rates tend to decrease in time. At 2, 12, 36 and 60 months from the initial TURB, these rates were 41%, 61%, 73% and 75% in the WLC arm versus 16%, 43%, 59% and 59% in the BLC arm [12]. In the future, this tendency should be evaluated during our medium and long–term follow-up.

Colombo states that the most important clinical impact of PDD was determined in cases of CIS, pT1G3 or multiple tumors. The method provides advantages concerning TURB efficacy and postoperative follow–up [13].

Burger et al. analyzed the treatment costs in bladder cancer, focusing on German patients. After an elaborate financial analysis, the authors concluded that PDD implied savings of 168 Euro per patient per year, during a follow–up period of 7 years [14]. So, concerns regarding the potential high costs of HAL–PDD seem to be unfounded.

## Conclusions

HAL fluorescence cystoscopy proved to be a powerful diagnostic method in cases of NIBT, emphasizing more effective imaging, higher detection rates, and improved sensitivity compared to standard cystoscopy.

Patients with Ta, T1 and especially CIS lesions were the main beneficiaries of this technique, as it provided them with better chances for a complete TURB. In this regard, BL–TURB may represent a superior therapeutic alternative, allowing a more precise delineation of the tumor margins.

The improved accuracy of BLC and the efficacy of BL–TURB led to a significantly reduced recurrence rate during the short–term follow–up.
